# ABA Represses the Expression of Cell Cycle Genes and May Modulate the Development of Endodormancy in Grapevine Buds

**DOI:** 10.3389/fpls.2017.00812

**Published:** 2017-05-19

**Authors:** Ricardo Vergara, Ximena Noriega, Karla Aravena, Humberto Prieto, Francisco J. Pérez

**Affiliations:** ^1^Laboratorio de Bioquímica Vegetal, Facultad de Ciencias, Universidad de ChileSantiago, Chile; ^2^Programa Doctorado en Ciencias Silvoagropecuarias y Veterinarias, Universidad de ChileSantiago, Chile; ^3^Laboratorio de Biotecnología Vegetal, Instituto de Investigaciones Agropecuarias, La PlatinaSantiago, Chile

**Keywords:** abscisic acid (ABA), cell cycle genes, endodormancy, grapevine buds

## Abstract

Recently, the plant hormone abscisic acid (ABA) has been implicated as a key player in the regulation of endodormancy (ED) in grapevine buds (*Vitis vinifera* L). In this study, we show that in the vine, the expression of genes related to the biosynthesis of ABA (*VvNCED1; VvNCED2*) and the content of ABA are significantly higher in the latent bud than at the shoot apex, while the expression of an ABA catabolic gene (*VvA8H3*) showed no significant difference between either organ. A negative correlation between the content of ABA and transcript levels of cell cycle genes (CCG) was found in both tissues. This result suggested that ABA may negatively regulate the expression of CCG in meristematic tissues of grapevines. To test this proposition, the effect of ABA on the expression of CCG was analyzed in two meristematic tissues of the vine: somatic embryos and shoot apexes. The results indicated that cell cycle progression is repressed by ABA in both organs, since it down-regulated the expression of genes encoding cyclin-dependent kinases (*VvCDKB1, VvCDKB2*) and genes encoding cyclins of type A (*VvCYCA1, VvCYCA2, VvCYCA3*), B (*VvCYCB*), and D (*VvCYCD3.2a*) and up-regulated the expression of *VvICK5*, a gene encoding an inhibitor of CDKs. During ED, the content of ABA increased, and the expression of CCG decreased. Moreover, the dormancy-breaking compound hydrogen cyanamide (HC) reduced the content of ABA and up-regulated the expression of CCG, this last effect was abolished when HC and ABA were co-applied. Taken together, these results suggest that ABA-mediated repression of CCG transcription may be part of the mechanism through which ABA modulates the development of ED in grapevine buds.

## Introduction

The central role of the plant hormone abscisic acid (ABA) in the control of seed dormancy is well established (Graeber et al., 2012). However, its role in the control of bud dormancy in woody perennials is not well defined. In poplar, the participation of ABA in the development of endodormancy (ED) induced by a short-day (SD)-photoperiod has been proposed, as both the content of ABA and ABA biosynthesis related genes peaked after 3–4 weeks of SD-treatment (Arora et al., 2003; Rohde and Bhalerao, 2007; Ruttink et al., 2007). Additionally, in parallel to increases in ABA content, several cell cycle genes (CCG) show decreased expression (Ruttink et al., 2007). However, because the content of ABA is very low when the dormant state is achieved (Ruttink et al., 2007), some doubts have been raised regarding the role of ABA in maintaining dormancy (Olsen, 2010).

In contrast to poplar and other tree species, *Vitis* does not set a terminal bud in response to SD-photoperiod, and the shoot apex does not enter ED, but the latent buds do (Kühn et al., 2009; Grant et al., 2013). During ED, the expression of ABA biosynthesis-related genes increased, while the expression of ABA catabolism-related genes decreased, and the content of ABA increased (Zheng et al., 2015). Exogenous application of ABA inhibited the release of buds from ED and attenuated the effect of the dormancy-breaking compound hydrogen cyanamide (HC), which promoted bud release (Zheng et al., 2015). Moreover, a SD photoperiod triggered the development of ED in grapevine buds (Kühn et al., 2009; Grant et al., 2013) and up-regulated the expression of ABA biosynthesis-related genes *VvNCED1* and *VvNCED2* (Parada et al., 2016). All this evidence supports the hypothesis that ABA promotes ED in grapevine buds. However, how this occurs is not well understood. ED, or winter rest, is characterized by reduced activity of the meristem of the latent bud and by the lack of response to growth-promoting stimuli (Rohde and Bhalerao, 2007). ABA and other hormones, such as gibberellins (GAs), auxin and cytokinin (CK) are well known to control the expression of CCG, which is certainly relevant in the development of ED (Campbell et al., 2014).

Progression of the cell cycle is primarily controlled by universally conserved cyclin-dependent kinases (CDKs) (Pines, 1995). Eight classes of CDKs were defined in *Arabidopsis* based on phylogenetic analyses (Vandepoele et al., 2002), but only the CDKA and CDKB groups have been well studied. CDKA is closely related to yeast Cdcd2/Cdc28 and to human CDK1, CDK2, and CDK3 (Hirt et al., 1992). CDKB is a plant-specific CDK that can be divided into two subgroups, CDKB1 and CDKB2. *CDKB1* transcripts accumulate during S, G2 and M phase, and in barley, this gene is down-regulated by ABA (Gendreau et al., 2012). *CDKB2* expression is specific to the G2 and M phases. CDK activity is negatively regulated by binding of the INHIBITOR OF CDK/KIP-RELATED PROTEIN (ICK) (De Veylder et al., 2001). ICK induces arrest or delay of the cell cycle in response to intra- or extracellular signals (Verkest et al., 2005). The activity and substrate specificity of CDKs are dependent upon their association with cyclins (Morgan, 1995). In plants, type A, B and D cyclins are thought to play a major role in cell cycle control (de Jager et al., 2001). The A- and B-type cyclins are expressed from S to M phase and control DNA replication, the G2/M transition and mitosis. The D-type cyclins are thought to be sensors of external signals and to play an essential role in cell cycle progression and in the entry of quiescent cells into the cell cycle (Kono et al., 2007). In the *Vitis* genome, the following cell cycle related genes have been identified: three CDKs, *VvCDKA, VvCDKB1* and *VvCDKB2*; two ICKs, *VvICK5, VvICK7*; two ICK-like genes, *VvICK3-like, VvICK7-like*; three type A cyclins, *VvCYCA1, VvCYCA2*, and *VvCYCA3*; one type B cyclin, *VvCYCB*; and three type D cyclins, *VvCYCD3.1, VvCYCD3.2a*, and *VvCYCD3.2b*.

In this study, we postulated that ABA, by repressing the expression of CCG, can modulate the development of ED in grapevine buds.

## Materials and Methods

### Plant Material

Latent buds and the shoot apexes from 8-year-old *Vitis vinifera* cv. Thompson seedless grown at the experimental station at the Chilean National Institute of Agriculture Research (INIA) in Santiago, Chile (33°34′S latitude) were collected on the same dates 7 March because it has been previously reported that the transition from paradormancy (PD) to ED occurs in mid-January (Kühn et al., 2009). Grape somatic embryos (GSE) used as plant material were initiated from a leaf disk of *in vitro* micro-propagated *Vitis vinifera* cv. Thompson seedless plantlets. NB2 culture medium (Dhekney et al., 2009) was used for callus micro-propagation, and cultures were maintained in the dark at 24°C until the formation of callus, when GSE were separated from the callus tissue and transferred to X6 medium and sub-cultured monthly. All samples were frozen in liquid N_2_ and stored at -80°C until used.

### ABA Treatment

For ABA treatment, GSE, the shoot apexes and latent buds of grapevines cv. Thompson seedless were sprayed with ABA (Sigma–Aldrich, United States) at concentrations of 10 and 100 μM, respectively, and water was used as a control. For GSE experiments, 0.3 g (0.1 g for each replicate) of GSE, mostly in the globular to heart-shaped stage, were transferred from subculture of semi-solid growth regulator-free X6 medium to liquid X6 medium. The effect of ABA (10 μM) on GSE was tested in a final volume of 30 ml of liquid X6 medium. Samples were shaken at 115 rpm for 72 h, and afterward, the liquid medium was discarded, and the GSE were frozen at –80°C until used. Shoot apexes were obtained from one month shoots from single-bud cuttings grown in a growth chamber and sprayed with 100 μM ABA. Samples were collected 24 h after the treatment, frozen in liquid N_2_ and stored at –80°C until used. Latent buds collected on March 23 were used to measure the content of ABA within the buds 48 h after the application of a 100 μM ABA solution, 2.5% HC solution and co-application of ABA and HC solutions.

### Hydrogen Cyanamide Treatment

The effect of the dormancy-breaking compound HC (Sigma–Aldrich, United States) on the expression of CCG and ABA metabolism-related genes was studied on single-bud cuttings of Thompson seedless grapevines. Canes collected on 8 June, 2015 were cut into single-bud cuttings. Cuttings (10–12 cm length) were sprayed with an aqueous solution containing 2.5% HC, and water was used as a control. Subsequently, the cuttings were placed in a growth chamber set at 23 ± 1°C with a 16 h photoperiod; 48 h after HC application, samples were taken for gene expression analysis.

### Extraction and Purification of ABA

Fresh plant material (10 buds and approximately 0.1 g of shoot apex for each biological replicate) was powdered in liquid nitrogen. The samples were extracted in a shaker for 1 h at 4°C and 10 min by ultra-sonication with 3 ml of 80% methanol containing 1% acetic acid and 3 ng of 2,3,5-triiodobenzoic acid (TIBA) as an internal standard (Sigma–Aldrich, United States). The extracts were centrifuged at 3000 × *g* for 10 min, and the supernatant was filtered through glass wool and a Sep-Pack C_18_ cartridge (Waters Assoc., Milford, MA, United States) that had been prewashed with 5 ml of 80% methanol. The procedure was repeated twice, and the filtrate was evaporated to dryness. After evaporation, the residue was dissolved in 1.2 ml ethyl acetate, and 1.2 ml of 0.5 M KH_2_PO_4_ pH 3.0 was added. The mixture was agitated with a vortex and centrifuged at 3000 rpm for 3 min. After centrifugation, the ethyl acetate layer was removed and placed in a clean tube. The aqueous phase was extracted once more with 1.2 ml of ethyl acetate. The collected ethyl acetate layers (2.4 ml) were evaporated to dryness. The samples were dissolved in 3 ml of ethylic ether and methylated with diazomethane in a diazomethane-generator apparatus according to manufacturer’s instructions. After methylation, the ether solution was evaporated to dryness, and the residue was dissolved in 50 μl methanol.

### Gas Chromatography Determination of ABA

A Shimadzu gas chromatograph, model GC-2014, equipped with an electron capture detector (ECD-2014, Shimadzu Corporation, Kyoto, Japan) and computer integrator was used for ABA determination (Vermeer et al., 1987). A CBP1 capillary column (25 m×0.25 mm I.D.) with helium as the carrier gas at a flux of 1.5 ml min^-1^ was used. The initial temperature of the column was 80°C and after 1 min was raised to 270°C at a rate of 20°C per min and maintained for 5 min. The injector was operated in the “splitless” mode at 225°C, and the temperature of the detector was 300°C. A calibration curve for ABA methyl ester and TIBA methyl ester was constructed. Endogenous ABA content was calculated from the peak area ratios of endogenous ABA to internal TIBA standard (Vermeer et al., 1987).

### RNA Purification and cDNA Synthesis

For gene expression analysis, total RNA was isolated and purified from GSE (0.1 g fr.wt), shoot apexes (0.1 g fr.wt) and latent buds (0.5 g fr.wt) of *Vitis vinifera* cv. Thompson seedless. In all cases, total RNA was extracted and purified using a modification of the method of Chang et al. (1993), as described in Noriega et al. (2007). DNA was removed by treatment with RNase-free DNase (1 U/μg) (Thermo Scientific, United States) at 37°C for 30 min. First-strand cDNA was synthesized from 1 μg of purified RNA with 1 μL oligo (dT)_12-18_ (0.5 μg × μL^-1^) as a primer, 1 μL dNTP mix (10 mM) and Superscript^®^ II RT (Invitrogen, CA, United States).

### Quantitative Real-Time PCR

Quantitative real-time PCR (RT-qPCR) was carried out in an Eco Real-Time PCR system (Illumina, Inc. San Diego, United States) using KAPA SYBR FAST (KK 4602) qPCR Master Mix (2 X). Primers for CDKs (*VvCDKA, VvCDKB1, VvCDKB2*), CDK inhibitors (*VvICK5, VvICK7*), ICK-like genes (*VvICK3-like*; *VvICK7-like*), type A cyclins (*VvCYCA1, VvCYCA2, VvCYCA3*), type B cyclins (*VvCYCB*), type D cyclins (*VvCYCD3.1, VvCYCD3.2a, VvCYCD3.2b*) and ABA metabolism-related genes (*VvNCED1, VvNCED2, VvA8H3*) were designed using the PRIMER3 software (Rozen and Skaletsky, 2000) (Supplementary Table S1). cDNA was amplified under the following conditions: denaturation at 94°C for 2 min and 40 cycles of 94°C for 30 s, 55°C for 30 s, and 72°C for 45 s. Relative changes in gene expression levels were determined using the 2^-ΔΔCT^ method (Livak and Schmittgen, 2001). Each reaction was performed in at least three biological replicates, each with three technical replicates. *VvUBIQUITIN* and/or *VvACTIN* were used as a reference gene for normalization

### Statistical Analysis

Differences in gene expression at different times were analyzed by ANOVA, and multiple comparison analysis was carried out by Dunnett’s and Tukey’s methods (α = 0.05). For pairwise comparisons, the Student’s *t*-test (α = 0.05) method was used.

## Results

### Expression of ABA Metabolism-Related Genes and ABA Content in Latent Buds and Shoot Apexes of Vines

**FIGURE 1:**
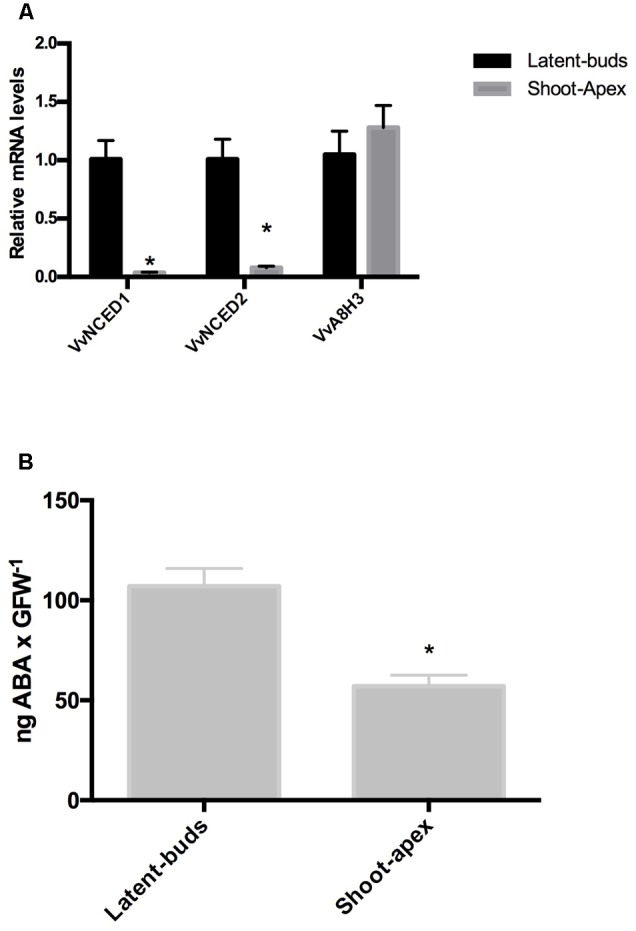
**Abscisic acid (ABA) content and ABA metabolism-related genes in the latent buds and shoot apexes of Thompson seedless grapevines.** Gene expression analysis of the ABA biosynthesis genes *VvNCED1* and *VvNCED2* and an ABA catabolism gene *VvA8H3* was performed by RT-qPCR and normalized against *VvUBIQUITIN* **(A)**. ABA content was determined as described in the Section “Materials and Methods” **(B)**. Values are the average of three biological replicates ± SD; (asterisk) Student’s *t*-test (α = 0.05).

In grapevines, transcript levels of the ABA biosynthesis genes *VvNCED1* and *VvNCED2* were significantly more highly expressed in the latent buds than at the shoot apexes (**Figure 1A**), while transcript levels of ABA catabolic gene *VvA8H3* showed no significant difference between either organ (**Figure 1A**). As expected, the content of ABA was higher in the latent buds than in the shoot apexes (**Figure 1B**).

### Expression of CCGs in the Latent Buds and Shoot Apexes of Vines

**FIGURE 2:**
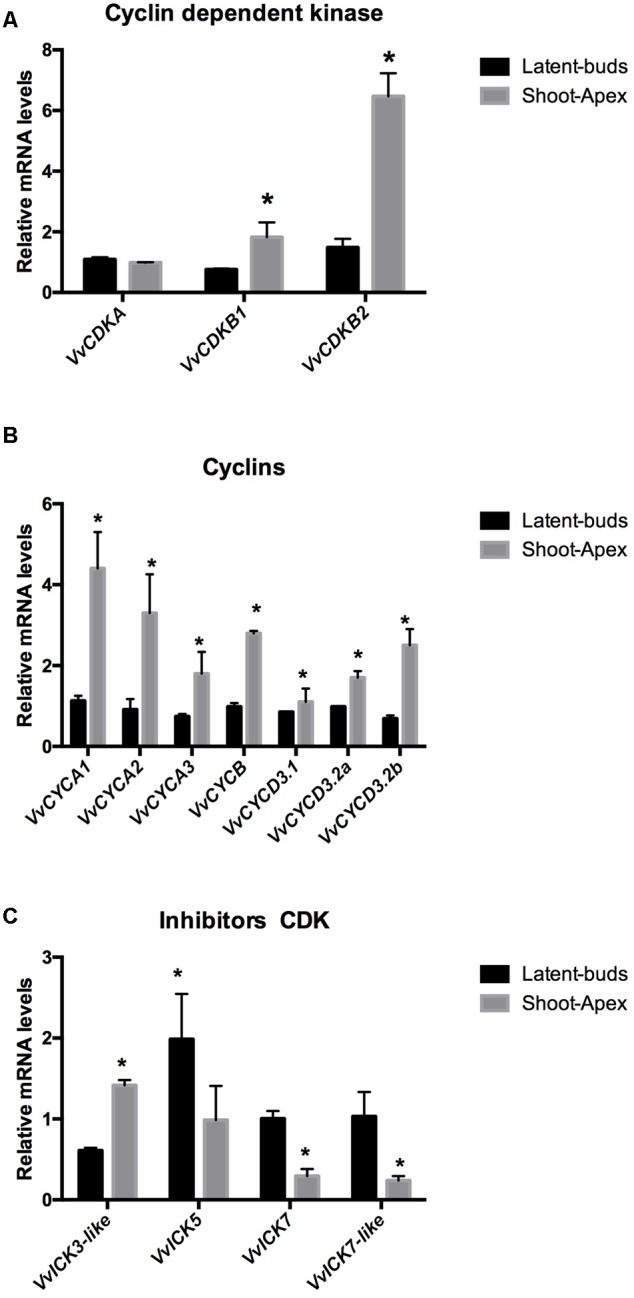
**Difference in gene expression of (A)** cyclin-dependent kinases (*VvCDKs*), **(B)** cyclins (*VvCYCs*), and **(C)** inhibitors of *CDKs* (*VvICKs*) in latent buds and shoot apexes. Gene expression analysis was performed by RT-qPCR and normalized against *VvUBIQUITIN*. Values are the average of three biological replicates with three technical repetitions ± SD; (asterisk) Student’s *t*-test (α = 0.05).

The following cell cycle-related genes have been identified in the *Vitis* genome: three CDK, *VvCDKA, VvCDKB1*, and *VvCDKB2*; two CDK inhibitors, *VvICK5, VvICK7*; two ICK-like genes, *VvICK3-like, VvICK7-like*; three type A cyclins, *VvCYCA1, VvCYCA2*, and *VvCYCA3*; one type B cyclin, *VvCYCB*; and three type D cyclins, *VvCYCD3.1, VvCYCD3.2a*, and *VvCYCD3.2b*. Transcript levels of CDKs *VvCDKB1* and *VvCDKB2* were expressed more highly at the shoot apex than at the latent bud, while *VvCDKA* was expressed similarly in both organs (**Figure 2A**). Expression analysis of type A cyclins, *VvCYCA1, VvCYCA2, VvCYCA3*; type B cyclin, *VvCYCB*; and type D cyclins, *VvCYCD3.1, VvCYCD3.2a, VvCYCD3.2b*, showed that all of them were expressed more at the shoot apex than at the latent buds (**Figure 2B**). In contrast, the transcript levels of the CDK inhibitors *VvICK5, VvICK7* and *VvICK7-like* were higher in the latent buds than at the shoot apexes, while *VvICK3-like* was expressed more at the shoot apex (**Figure 2C**).

### ABA Down-regulated the Expression of CCGs in GSEs

**FIGURE 3:**
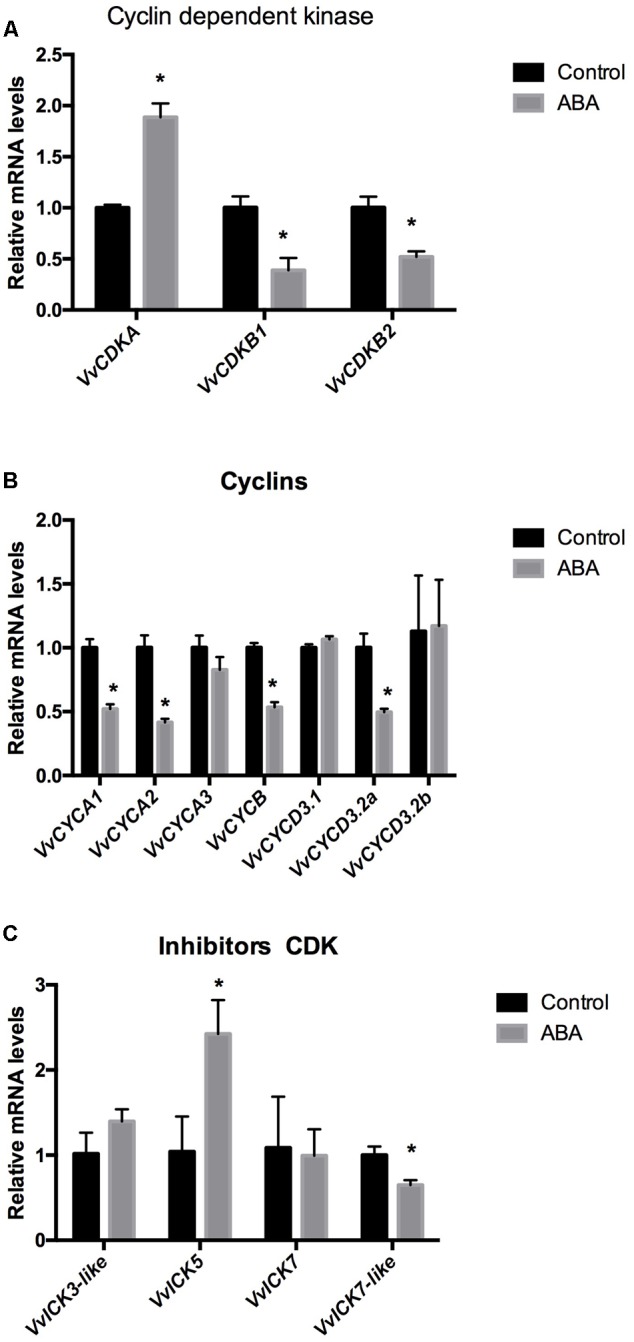
**Effect of ABA on the expression of (A)** cyclin-dependent kinases (*VvCDKs*), **(B)** cyclins (*VvCYCs*) and **(C)** inhibitors of *CDKs* (*VvICKs*) on Thompson seedless somatic embryos.Gene expression analysis was performed by RT-qPCR and normalized against *VvACTIN*. Values are the average of three biological replicates with three technical repetitions ± SD; (asterisk) Student’s *t*-test (α = 0.05).

To verify whether ABA affects the expression of CCGs in meristematic tissues of grapevines, we analyzed its effect on the expression of the CDKs *VvCDKA, VvCDKB1, VvCDKB2* (**Figure 3A**), the type A cyclins *VvCYCA1, VvCYCA2, VvCYCA3*, the type B cyclin *VvCYCB*, and the type D cyclins *VvCYCD3.1, VvCYCD3.2a, VvCYCD3.2b* (**Figure 3B**) and on the CDK inhibitors *VvICK5* and *VvICK7* and two ICK-like genes, *VvICK3-like* and *VvICK7-like* genes (**Figure 3C**) in GSE. The results indicated that ABA inhibits cell cycle progression since most of the genes coding for CDKs (*VvCDKs*) and for cyclins (*VvCYCs*) were down-regulated by the plant hormone (**Figures 3A,B**), while the expression of a CDK inhibitor (*VvICK5*) was up-regulated (**Figure 3C**). Similar results were obtained when ABA was applied to other organs containing meristems, such as the shoot-apex (**Supplementary Figure S1**).

### Changes in the Expression of CCG during ED in Grapevine Buds

**FIGURE 4:**
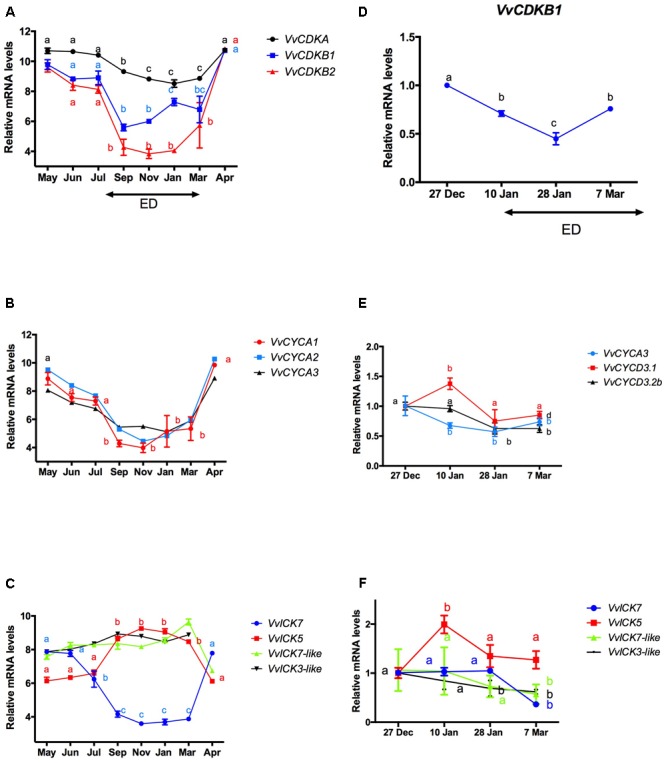
**Changes in the expression of (A)** cyclin-dependent kinases (*VvCDKs*), **(B)** cyclins (*VvCYCs*), and **(C)** inhibitors of *CDKs* (*VvICKs*) during the development of endodormancy (ED) in Tempranillo grapevine buds grown at the Northern Hemisphere and in Thompson seedless grapevine buds grown in the Southern Hemisphere **(D–F)**. Values for Tempranillo grapevine buds were taken from (Díaz-Riquelme et al., 2012), and those for Thompson seedless were determined by RT-qPCR and normalized against *VvUBIQUITIN*. Values for Tempranillo and Thompson seedless are the average of three biological replicates with three technical repetitions ± SD. Different lower case letters represent significant differences between transcript collected from grapevine buds at different times Tukey’s test (α = 0.05).

It has been reported that the content of ABA increases with the development of ED in grapevine buds (Zheng et al., 2015). To test whether this increase is consistent with a decrease in the expression of CCG, transcript levels of CCG taken from microarray data on the development of Tempranillo grape buds grown in the Northern Hemisphere (Díaz-Riquelme et al., 2012) were plotted throughout the ED period (**Figures 4A–C**). Furthermore, to verify the data of Díaz-Riquelme et al. (2012), we used RT-qPCR to analyze the transcript levels of CCG in buds of Thompson seedless grapevines grown in the Southern Hemisphere (**Figures 4D–F**). The results showed that in either cases, the expression of *VvCDKs* (**Figures 4A,D**) and *VvCYCs* (**Figures 4B,E**) decreased with the development of the ED, while the expression of *VvICK5* increased, and the expression of *VvICK7* decreased while *VvICK7*-like and *VvICK3*-like remained relatively unchanged (**Figures 4C,F**).

### Hydrogen Cyanamide Reduced the Content of ABA in Grapevine Buds

**FIGURE 5:**
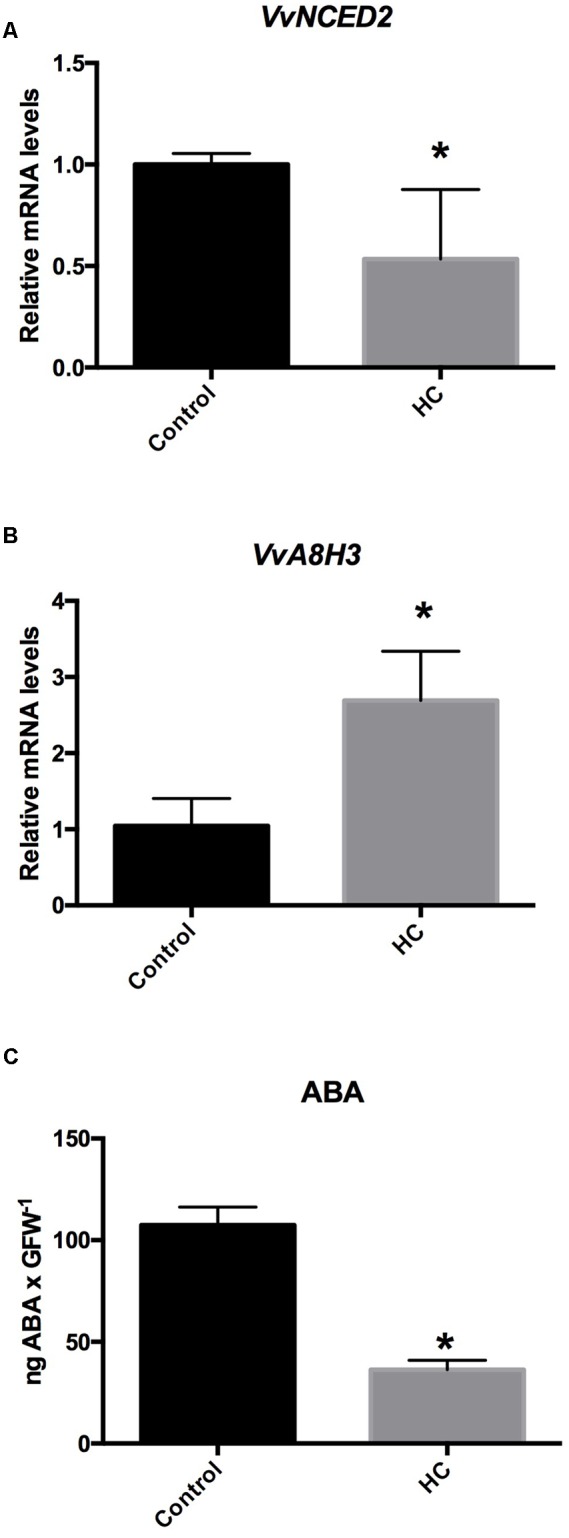
**Hydrogen cyanamide (HC) altered the expression of ABA metabolism-related genes (A,B)** and reduced the content of ABA in grapevine buds **(C)**. HC down-regulated the expression of the ABA biosynthesis gene *VvNCED2* **(A)** and up-regulated the expression of the ABA catabolism-related gene *VvA8H3* **(B)** and reduced the content of ABA **(C)** in Thompson seedless grapevine buds. Gene expression analysis was performed by RT-qPCR, values were normalized against *VvUBIQUITIN*, and the content of ABA was determined as described in the Section “Materials and Methods”. Values are the average of three biological replicates with ± SD; (asterisk) Student’s *t*-test (α = 0.05).

To test whether HC reduces the content of ABA in grapevine buds, single-bud cuttings of cv. Thompson seedless were sprayed with a 2.5% solution of HC, and 48 h after treatment, analysis was conducted for the ABA biosynthesis-related gene *VvNCED2*, the ABA catabolism-related gene *VvA8H3* and ABA content. The results showed that HC down-regulated *VvNCED2* (**Figure 5A**) and up-regulated *VvA8H3* gene expression (**Figure 5B**) and ABA content was drastically reduced (**Figure 5C**).

### Hydrogen Cyanamide Up-regulated the Expression of CCG

**FIGURE 6:**
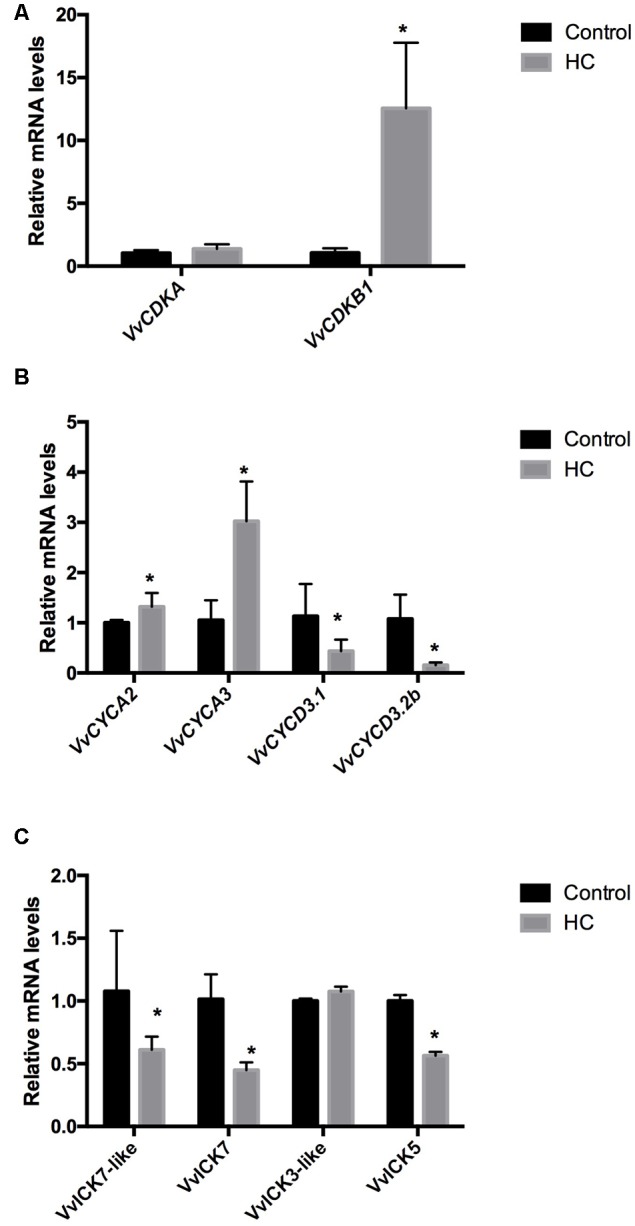
**Effect of HC on the expression of (A)** cyclin-dependent kinases (*VvCDKs*), **(B)** cyclins (*VvCYCs*), and **(C)** inhibitors of *CDKs* (*VvICKs*) in Thompson seedless grapevine buds. Gene expression analysis was performed by RT-qPCR and normalized against *VvUBIQUITIN*. Values are the average of three biological replicates with three technical repetitions ± SD; (asterisk) Student’s *t*-test (α = 0.05).

To test whether HC up-regulates the expression of CCG in grapevine buds, single-bud cuttings of cv. Thompson seedless were sprayed with a 2.5% solution of HC, and 48 h after treatment, gene expression analysis was conducted. The results showed that HC up-regulated the expression of the CDK *VvCDKB1* (**Figure 6A**) and of the A-type cyclins *VvCYCA2* and *VvCYCA3*; however, the expression of *VvCYCDs* was inhibited (**Figure 6B**), and the expression of CDK inhibitors, *VvICK5* and *VvICK7*, was down-regulated (**Figure 6C**).

### Incorporation of ABA into Grapevine Buds

**FIGURE 7:**
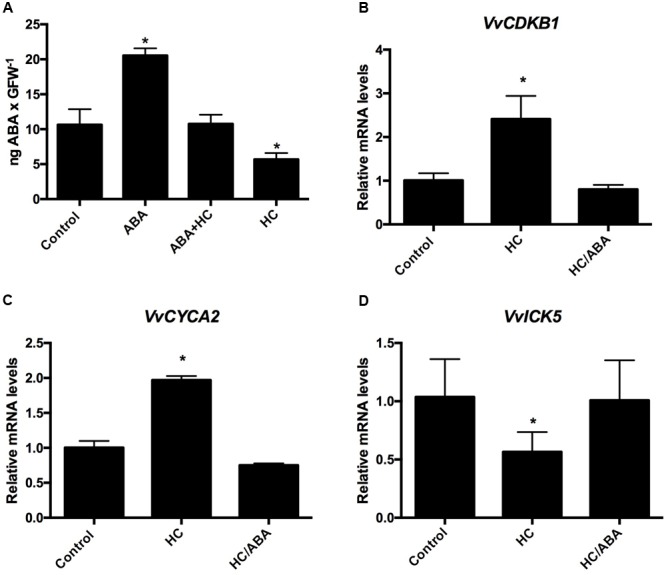
**Incorporation of ABA into grapevine buds **(A)**.** Co-applications of HC and ABA on the expression of CCG in grapevine buds **(B–D)**. The content of ABA was determined in latent grapevine buds collected on March 23 after the application of the following solutions 100 μM ABA, 100 μM ABA + 2.5% HC, 2.5% HC and water as control. Values are the average of three biological replicates ± SD; (asterisk) represents significance difference with respect to the control (Dunnett’s multiple comparison test *p* < 0.05). Single-bud cuttings of Thompson seedless grapevine collected on March 23 were sprayed with the following solutions 2.5% HC, 2.5% HC + 100 μM ABA and water as control and 48 h after treatment gene expression analysis was performed by RT-qPCR and normalized against *VvUBIQUITIN*. Values are the average of three biological replicates with three technical repetitions ± SD; (asterisk) represents significance difference with respect to the control (Dunnet’s multiple comparison test *p* < 0.05).

To determine the content of ABA within the buds after spraying them with the following solutions; 100 μM ABA, 100 μM ABA + 2.5% HC, 2.5% HC, single-bud cuttings harvested on March 23 were sprayed with the above solutions and water as control, 48 h after the treatment, analysis was conducted to determine the ABA content. The results indicated that the application of 100 μM ABA doubled the concentration of ABA in relation to control buds, while the application of HC, reduced the concentration of ABA to half of the concentration of the control, and co-application of ABA and HC resulted in ABA content similar to control buds (**Figure 7A**). To test if the modifications produced by HC on the expression of CCG are due to their reducing effect on the ABA content, single-bud cuttings of grapevines were sprayed with the following solutions 2.5% HC; 2.5% HC + 100 μM ABA and water as control. Expression of genes that were up-regulated by HC were analyzed 48 h after treatment. The results showed that transcript levels of *VvCDKB1, VvCYCA3*, and *VvICK5* remained similar to control buds after the co-application of HC and ABA (**Figures 7B–D**).

## Discussion

Abscisic acid has been shown to inhibit DNA replication in root tips and embryos of *Fraxinus excelsior* (Van Overbeek et al., 1967; Villiers, 1968). It has also been described as an inhibitor of cell division and/or DNA synthesis in different plant cell types (Newton, 1977; Robertson et al., 1990). Using a construct in which expression of the ß-glucuronidase (GUS) reporter gene was driven by the *CDKA* promoter, Hemerly et al. (1993) demonstrated that ABA inhibits promoter activity in the root. On the other hand, ABA induced the expression of the cyclin-dependent protein kinase inhibitor (*ICK/KRP*) (Wang et al., 1981), and it has been suggested that ABA blocks cell division at the G1/S transition by increasing the levels of ICK/KRP (del Pozo et al., 2005). In rice, it has been demonstrated that ABA inhibited shoot growth and induced expression of *OsKRP4, OsKRP5*, and *OsKRP6* (Meguro and Sato, 2014).

The transition of bud from a state of dormancy to more active growth has been associated with increased expression of genes that regulate or are involved in the cell cycle (Devitt and Stafstrom, 1995; Shimizu and Mori, 1998; Kebrom et al., 2010). It has been shown that ABA restricts lower bud outgrowth and promotes correlative inhibition in *Arabidopsis* axillary buds (Yao and Finlayson, 2015). Moreover, these authors showed that CCGs *CYCLIN A2;1* and *PROLIFERATING CELL NUCLEAR ANTIGEN1* (*PCNA1*) were suppressed by ABA, suggesting that ABA may inhibit bud growth in part by suppressing elements of the cell cycle machinery.

In this study, the negative relationship found between the content of ABA and the expression level of the CCG at the shoot apex and the latent buds of grapevines, prompted us to investigate the effect of ABA on the expression of CCG in grapevine buds. The fact that ABA down-regulated the expression of genes encoding type B CDKs (*VvCDKBs*) and cyclins (*VvCYCs*) and up-regulated the expression of a gene encoding a CDK inhibitor (*VvICK5*), concurrently with the fact that the concentration of ABA within buds falls within a physiological range after the exogenous application of a 100 μM ABA solution, suggests that by modulating the expression of the CCG, ABA could arrests the cell-cycle progression and cell division in meristematic tissues of grapevine. Recently, it has been shown that exogenous application of ABA delays the sprouting of buds and attenuates the effect of HC on promoting bud break in single-bud cuttings of grapevines (Zheng et al., 2015). Likewise, the fact that HC besides reducing the content of ABA, up-regulates the expression of CCGs (*VvCDKB1, VvCYCA3*) and down-regulates the expression of cell cycle inhibitors (*VvICK5* and *VvICK7*) suggests that the dormancy breaking effect of HC could be mediated by ABA. In support of this, co-applications of HC and ABA to grapevine buds reduced significantly the expression of CCG relative to HC-treated buds, validating thus the statement that the up-regulation of CCG by HC is mediated by a reduction in ABA content. Other evidence that support the role of ABA in the acquisition and maintenance of ED in grapevine buds is that the SD-photoperiod up-regulates the expression of ABA biosynthetic genes (*VvNCED1, VvNCED2*) (Parada et al., 2016) and simultaneously triggers ED in grapevine buds (Kühn et al., 2009; Grant et al., 2013). Interestingly, when transcript levels of CCG obtained from microarray analysis for bud development of the grape variety Tempranillo grown in the Northern Hemisphere (Díaz-Riquelme et al., 2012) were plotted throughout ED, the expression of genes encoding CDKs *VvCDKA, VvCDKB1*, and *VvCDKB2* and cyclins *VvCYCAs* was down-regulated, while the expression of the CDK inhibitor *VvICK5* was up-regulated. This finding agrees with our results obtained with Thompson seedless grapevine buds grown in the Southern Hemisphere. Moreover, the increases in the content of ABA detected by Zheng et al. (2015) throughout ED in grapevine buds is consistent with results showing a decrease in the expression of CCG, since during ED the bud meristem remains inactive due to the arrest of the cell cycle. Interestingly, HC up-regulated the expression of *VvCDKB1* and *VvCYCAs* but down-regulated the expression of *VvCYCDs*. The D-type cyclins are thought to be sensors of external signals and to play an essential role in the entry of quiescent cells into the cell cycle (Kono et al., 2007), while type A and B cyclins and the CDK *VvCDKB1* are expressed during the transition from S to M phase and control DNA replication, the G2/M transition, and mitosis. In a previous study, we found that ABA inhibits respiration in dormant grapevine buds (Parada et al., 2016), and in this work, we found that ABA inhibits cell-cycle progression. Therefore, the question that emerges is if there is a relationship between mitochondrial respiration and cell-cycle progression in grapevine buds. In mammals, it has been reported that the cyclin B1/Cdk1 complex is capable of increasing mitochondrial respiration, with enhanced oxygen consumption and ATP generation during the G2/M transition phase of the cell cycle by phosphorylating a cluster of mitochondrial proteins, including the complex I subunits in the respiratory chain (Wang et al., 2014). Although this interaction has not been reported in plants, it suggests that ABA may be a master regulator of ED in grapevine buds since the repression of CCG expression could reduce mitochondrial respiration, thus causing the arrest of meristem activity in grapevine buds.

## Author Contributions

RV: planification and revision of the manuscript and performed most of the experiments. XN: performed RT-qPCR experiments. KA: performed ABA determinations. HP: collaborated with experiments carried-out in GSE. FP: planning, design of experiments, and writing of the manuscript.

## Conflict of Interest Statement

The authors declare that the research was conducted in the absence of any commercial or financial relationships that could be construed as a potential conflict of interest.

## Supplementary Material

FIGURE S1 **Effect of ABA on the expression of (A)** cyclin-dependent kinases (*VvCDKs*), **(B)** cyclins (*VvCYCs*), and **(C)** inhibitors of CDKs (*VvICKs*) on the shoot-apex of Thompson seedless grapevines. Gene expression analysis was performed by RT-qPCR and normalized against *VvACTIN*. Values are the average of three biological replicates with three technical repetitions.
