# Supplementary material for: ABA Represses the Expression of Cell Cycle Genes and May Modulate the Development of Endodormancy in Grapevine Buds
Source: Front Plant Sci. 2017 May 19;8:812. doi: 10.3389/fpls.2017.00812 (PMC5437152; doi:10.3389/fpls.2017.00812)
Supplement: Supplementary file 1 [file Table_1.DOC]

| **Genes** | **Forward** | **Reverse** |
| --- | --- | --- |
| ***VvICK7-like* (GSVIVT01022145001)** | 5'GCTCAGGGACAGTGAAGAGG 3´ | 5´CCACCACTCGTCCAGAATTT 3´ |
| ***VvICK3-like* (GSVIVT01028640001)** | 5´CAGAGGCTCCAGAAAACGAC 3´ | 5´TTGAGCTCGCCTTAGGGTTA 3´ |
| ***VvICK5* (GSVIVT01021078001)** | 5´AAGAGGTGAGGTTGCGGTTA 3´ | 5´GCTAGAGCTTGCCTGCTGTT 3´ |
| ***VvICK7* (GSVIVT01031765001)** | 5´ACGTCGCCTTCGTCTAATGT 3´ | 5´CACCGAGAACTTGCTCAACA 3´ |
| ***VvCDKA* (GSVIVT01026700001)** | 5´TTGGTGAAGGAACCTATGGTG 3´ | 5´CAAGCGGATCTTCTTCAAGG 3´ |
| ***VvCDKB1* (GSVIVT01029843001)** | 5´GGTTTACAAGGCCAAGGACA 3´ | 5´GACTGGGAGAGCATCTGGAG 3´ |
| ***VvCDKB2* (GSVIVT01013440001)** | 5´CTGGAGAAGGTAGGGGAAGG3´ | 5´AACATGCGAAGCAAGGAGAC 3 |
| ***VvCYCA1* (GSVIVT01008823001)** | 5´GCTATGTCGACCCAGAATCG 3´ | 5´GCCGAGCTTTTTAACCCACT 3´ |
| ***VvCYCA2* (GSVIVT01035718001)** | 5´GCGAGCTAAAAGAGGTCCTTC 3´ | 5´TTTTGGCATCCTCTTGAACC 3 |
| ***VvCYCA3* (GSVIVT01009399001)** | ´5´GATTTTGGTGGATTGGTTGG 3´ | 5´ACCCAATAGCTGGAGCCTCT 3´ |
| ***VvCYCD3.1* (GSVIVT01009432001)** | 5´ACCTCCTATGCGAAGACGAA3´ | 5´TGCTTTCGGGGTTACTATGG3´ |
| ***VvCYCD3.2a* (GSVIVT01003175001)** | 5´AGTCACCCCACTCTCCTTCA3´ | 5´AGGAGAAAACGCTCGCATAG3´ |
| ***VvCYCD3.2b* (GSVIVT01000226001)** | 5´GGGTTTTCTGCTCTGACTGC3´ | 5´GAAGAGGAACATCGGTCTCG3´ |
| ***VvNCED2* (GSVIVT01038080001)** | 5´TTTGTGGCACGACGAGAAGAC3´ | 5´AGGGAACTCGTGAGGGAAGT3´ |
| ***VvNCED1* (GSVIVT01029057001)** | 5´CAAATGCTGGCCTCGTCTAT3´ | 5´GCCTTTGATCTTCACGTGGT 3´ |
| ***VvA8H3* (GSVIVT01009493001)** | 5´CCCACCACCTAGTGACCAAG 3´ | 5´TTCACAGGGACTGGGAATG 3´ |
| ***VvUBQ* (GSVIVT01038617001)** | 5´CAGCACCGACAAAGACTTGA3´ | 5´CCCAGTTAGGGTTTTCACGA3´ |
| ***VvACT* (GSVIVT01026580001)** | 5´TTTTAATGTGCCTGCCATGT 3´ | 5´AGCATGGGGAAGTGCATAAC 3´ |

**Suppl. Tab. S1.** Primers used for RT-qPCR
