# Supplementary figures and images for: ABA Represses the Expression of Cell Cycle Genes and May Modulate the Development of Endodormancy in Grapevine Buds
Source: Front Plant Sci. 2017 May 19;8:812. doi: 10.3389/fpls.2017.00812 (PMC5437152; doi:10.3389/fpls.2017.00812)

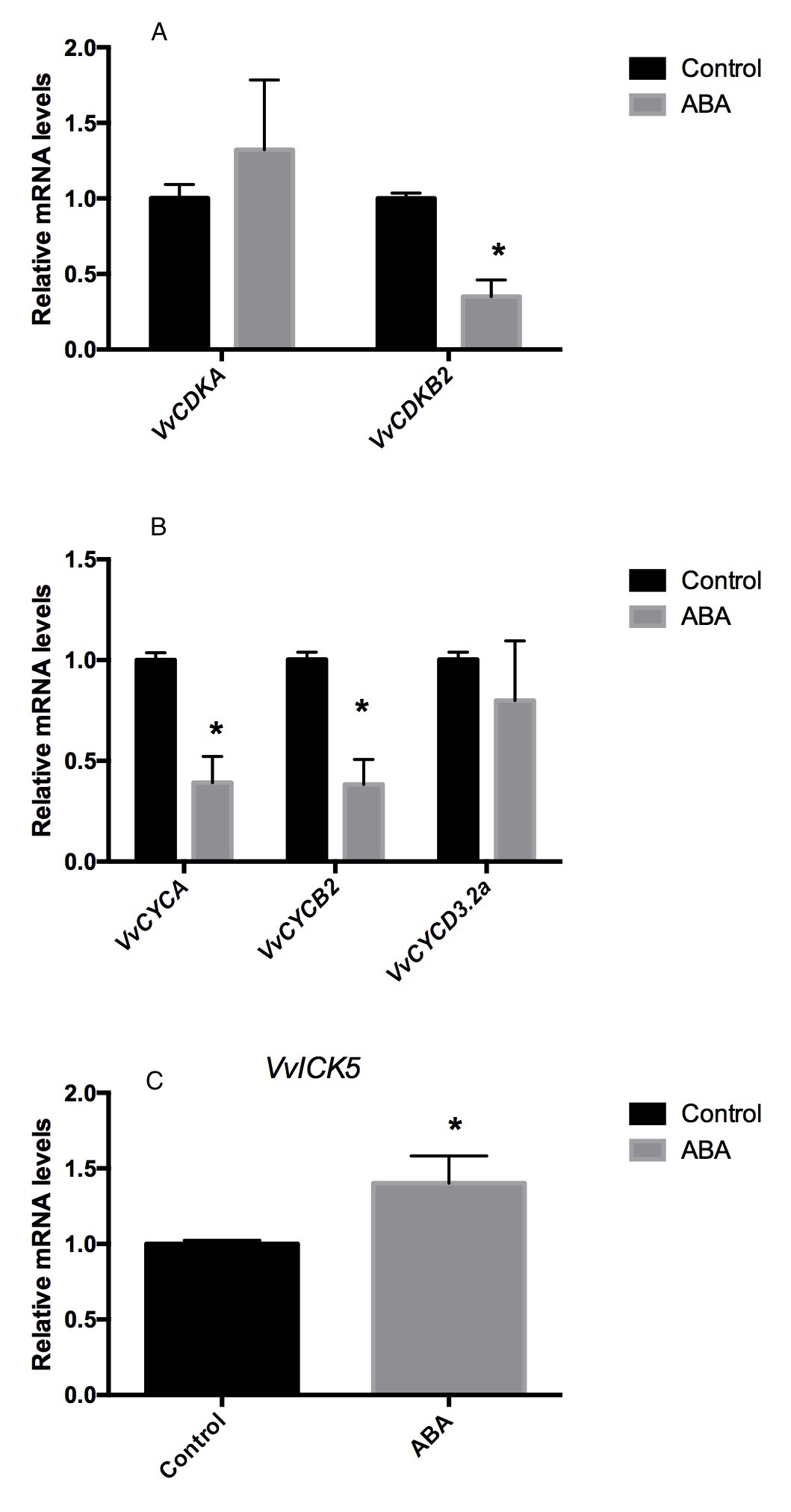

Supplement: FIGURE S1 — Effect of ABA on the expression of (A) cyclin-dependent kinases (VvCDKs), (B) cyclins (VvCYCs), and (C) inhibitors of CDKs (VvICKs) on the shoot-apex of Thompson seedless grapevines. Gene expression analysis was performed by RT-qPCR and normalized against VvACTIN. Values are the average of three biological replicates with three technical repetitions. [file Image_1.jpg]
